# Contrast-free high-resolution 3D magnetization transfer imaging for simultaneous myocardial scar and cardiac vein visualization

**DOI:** 10.1007/s10334-020-00833-9

**Published:** 2020-02-20

**Authors:** Karina López, Radhouene Neji, Rahul K. Mukherjee, John Whitaker, Alkystis Phinikaridou, Reza Razavi, Claudia Prieto, Sébastien Roujol, René Botnar

**Affiliations:** 1 School of Biomedical Engineering and Imaging Sciences, King’s College London, St Thomas’ Hospital, 3rd Floor Lambeth Wing, London, SE1 7EH UK; 2 MR Research Collaborations, Siemens Healthcare Limited, Frimley, UK

**Keywords:** Fibrosis, Coronary vessels, Magnetic resonance imaging

## Abstract

**Objective:**

To develop a three-dimensional (3D) high-resolution free-breathing magnetization transfer ratio (MTR) sequence for contrast-free assessment of myocardial infarct and coronary vein anatomy.

**Materials and methods:**

Two datasets with and without off-resonance magnetization transfer preparation were sequentially acquired to compute MTR. 2D image navigators enabled beat-to-beat translational and bin-to-bin non-rigid motion correction. Two different imaging sequences were explored. MTR scar localization was compared against 3D late gadolinium enhancement (LGE) in a porcine model of myocardial infarction. MTR variability across the left ventricle and vessel sharpness in the coronary veins were evaluated in healthy human subjects.

**Results:**

A decrease in MTR was observed in areas with LGE in all pigs (non-infarct: 25.1 ± 1.7% vs infarct: 16.8 ± 1.9%). The average infarct volume overlap on MTR and LGE was 62.5 ± 19.2%. In humans, mean MTR in myocardium was between 37 and 40%. Spatial variability was between 15 and 20% of the mean value. 3D whole heart MT-prepared datasets enabled coronary vein visualization with up to 8% improved vessel sharpness for non-rigid compared to translational motion correction.

**Discussion:**

MTR and LGE showed agreement in infarct detection and localization in a swine model. Free-breathing 3D MTR maps are feasible in humans but high spatial variability was observed. Further clinical studies are warranted.

**Electronic supplementary material:**

The online version of this article (10.1007/s10334-020-00833-9) contains supplementary material, which is available to authorized users.

## Introduction

MRI plays an important role for non-invasive assessment of myocardial fibrosis in patients with ischemic and non-ischemic cardiomyopathies. Myocardial scar assessment has been shown to have important prognostic value [1–3]. Furthermore, in patients requiring cardiac resynchronization therapy, detection of focal fibrosis and coronary sinus/vein anatomy may help to guide the intervention [4]. In these applications, the accurate discrimination of fibrotic or scarred tissue from surrounding healthy myocardium is crucial.

Late gadolinium enhancement (LGE) MRI is the gold standard for the assessment of myocardial fibrosis [5]. LGE shows enhanced signal intensity by targeting the enlarged extracellular space in fibrotic tissue using gadolinium-based contrast agents [6]. LGE is a qualitative approach where the time point of imaging after the injection of a contrast agent determines the pattern of enhancement. While core infarct regions will enhance strongly and at a late stage during the contrast washout (> 25 min), the signal from the rim of an infarcted region (area at risk) might enhance very early on (2–3 min), only to return to normal at the late stage [7]. Furthermore, the use of gadolinium-based contrast agents may generate accumulation in the neural system [8] and/or adverse contrast reaction. Therefore, the development of a semi-quantitative and gadolinium-free approach for the assessment of myocardial fibrosis may be of great interest.

Magnetization transfer (MT) is an endogenous MR contrast mechanism, which does not require the injection of a contrast agent. MT has shown to be sensitive to structural changes associated with myocardial fibrosis, such as the increase in collagen content [9–11]. MT was first demonstrated by Wolff and Balaban in kidney and skeletal muscle [12], showing a signal decrease from free water protons, after selectively saturating a pool of protons with restricted motion with an off-resonance preparation. The signal decrease is explained by an exchange of magnetization between the water protons (free pool) and those with restricted motion (bound pool). The characteristics of the exchange are determined by the tissue composition, in particular, the size and distribution of its macromolecular or bound pool component.

A common application of MT contrast has been to suppress signal coming from protein-rich tissue, such as white matter or muscle [13, 14]. This effect can generate good contrast between myocardium and blood irrespective of blood oxygenation and therefore improve cardiac vein visualization [15]. While *T*_2_ preparation is known to be useful in coronary angiography [16], the large reduction in *T*_2_ of deoxygenated blood causes difficulty in coronary vein imaging. MT preparation is largely independent of the magnetic susceptibility effect that causes *T*_2_ reduction in venous blood and has been shown to generate a contrast enhancement that is superior to *T*_2_-based approaches in the cardiac veins [17].

Another application of MT in cardiac MRI is the semi-quantitative magnetization transfer ratio (MTR). In MTR, two images are acquired, with and without MT preparation, and the percentage difference or ratio between them is calculated. A breath-held 2D cine steady state technique showed reduced MTR values associated with LGE enhancement in acute myocardial infarction (MI) patients [18]. However, breath-holding can be difficult in sick patients, where the length of breath-hold may limit the spatial resolution and different breath-hold positions may lead to misregistration artefacts between slices. Germain et al. has recently proposed a diaphragmatic navigated free-breathing 3D on-resonance MT approach [19], where they show some agreement between LGE enhancement and reduced MT effects in patients with acute or recent MI. However, partial volume effects and likely residual motion seem to have caused significant intra- and intersubject variability. We hypothesize that a significant increase in slice resolution and two-dimensional navigation may have a good impact in reproducibility and accuracy.

To address the above challenges, we developed and evaluated a novel free-breathing motion corrected 3D whole-heart MTR sequence for the simultaneous assessment of localized fibrosis and coronary vein anatomy without the administration of a contrast agent.

## Methods

### Sequence acquisition and reconstruction

A prototype motion-corrected free-breathing 3D magnetization transfer contrast imaging sequence was implemented on a 1.5 T MR scanner (MAGNETOM Aera, Siemens Healthcare, Erlangen, Germany). The sequence comprised two sequentially acquired 3D datasets, one with MT preparation and one without any preparation (hereon dataset REF).

**Fig. 1:**
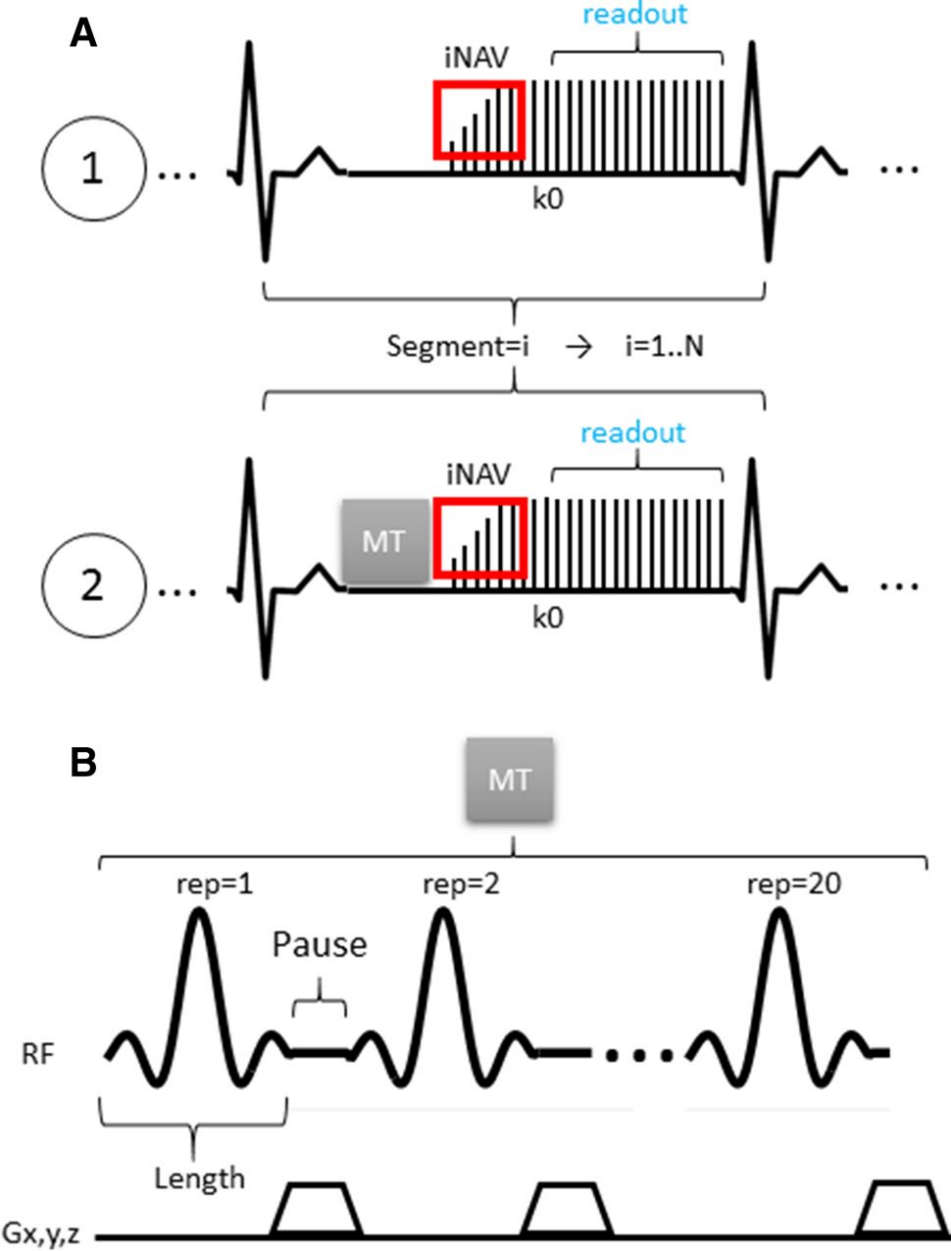
**a** Acquisition scheme for the two 3D segmented (electrocardiogram-triggered) datasets (with bSSFP), the reference (REF) dataset (without preparation) and the MT-prepared dataset (with MT preparation) are acquired sequentially. Both acquisitions include 2D image navigators (iNAVs) obtained by encoding the first 14 start-up pulses of the imaging module. **b** Scheme of the MT preparation module which consisted of 20 off-resonance pulses (20 ms) separated by pause periods (1.5 ms) where 3D spoiler gradients were played out

A pulse diagram of the sequence is shown in Fig. 1a. Each acquisition was performed with a Cartesian trajectory with spiral profile order [20] and preceded by a 2D image navigator (iNAV) [21] to enable beat-to-beat translational motion correction in foot-to-head and left-to-right directions. The iNAVs were generated by spatially encoding the first 14 (start-up) pulses of the imaging sequence. The high-resolution 3D datasets were acquired in segmented fashion over multiple heartbeats, and data sampling started with the 15th RF pulse to the end of the echo train in every heartbeat. The spiral-profile Cartesian trajectory was chosen because it produces incoherent and noise-like undersampling artefacts which are beneficial for undersampled reconstruction. In addition, it provides some motion robustness associated with spiral sampling while maintaining a Cartesian computationally efficient reconstruction.

Spoiled gradient echo (SPGR) and balanced steady-state free-precession (bSSFP) imaging modules were investigated with the described MT preparation module. Data sampling was made to coincide with the diastolic resting period to minimize the impact of cardiac motion. The subject-specific trigger delay time and data acquisition window were determined using a free-breathing 2D cine, acquired in transversal orientation prior to the proposed sequence.

The MT preparation (represented in Fig. 1b) consisted of a train of 20 Sinc-shaped off-resonance RF pulses with individual length of 20.48 ms, separated by 1.5 ms pause periods. The preparation train was played out immediately before the imaging module in every heartbeat (or segment) of the MT-prepared image. The off-resonance frequency and flip angle of the pulses were optimized as described in the following sections.

**Fig. 2:**
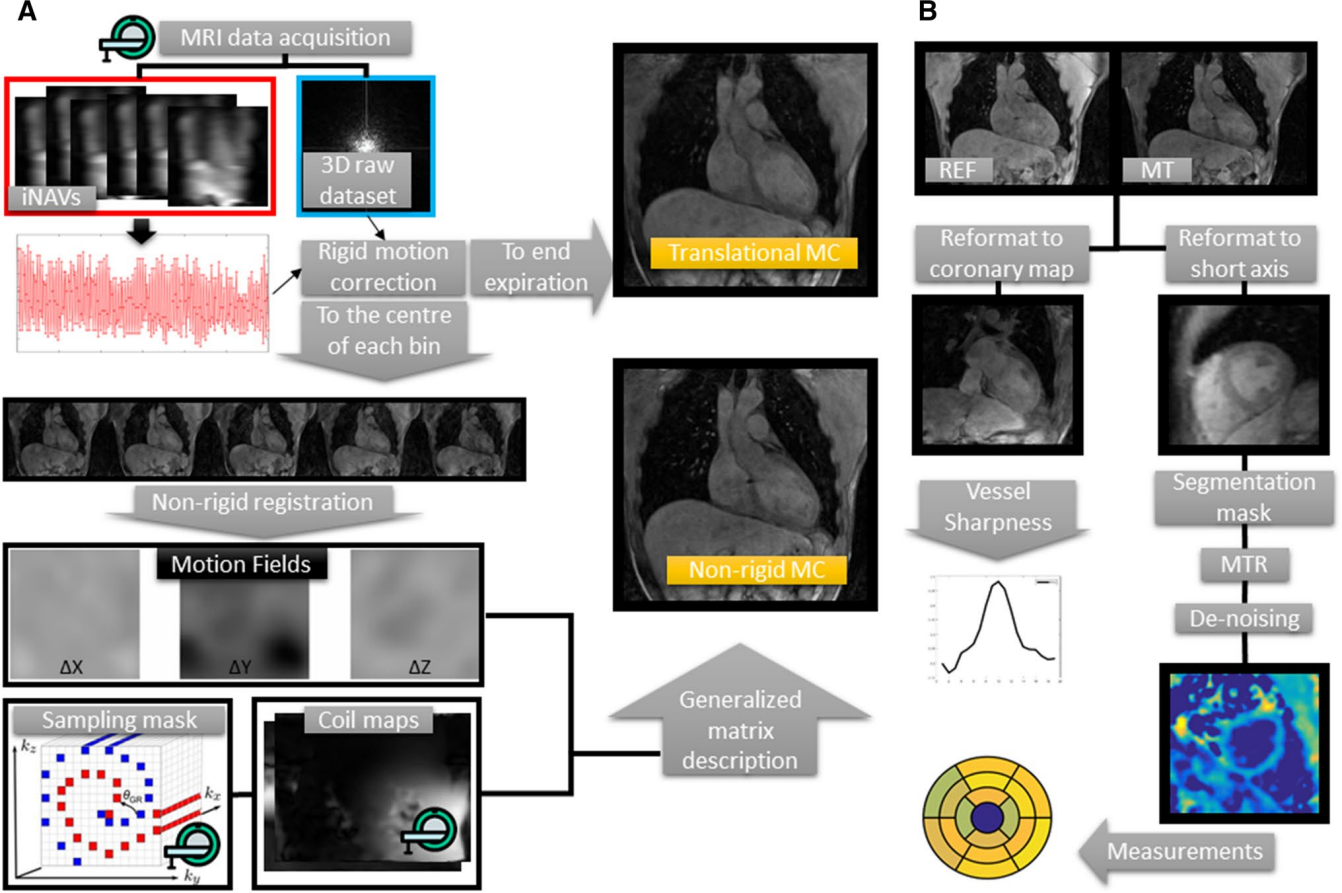
**a** Data flow during image acquisition and reconstruction. From the top left, raw data and image navigators (iNAVs) were obtained from the scanner; iNAVS were used for translational motion correction in two ways: (1) to the end expiration phase for translational motion correction (MC); (2) to the centre of each respiratory phase (or bin), followed by non-rigid registration of the bins and generation of 3D motion fields. Non-rigid MC was performed using a generalized matrix description that included the calculated motion fields, sampling mask and coil maps. **b** Data flow during image analysis. MT and REF datasets were reformatted to either a 2D coronary projection image for vessel sharpness calculation, or to left-ventricle short axis view for manual segmentation and creation of a mask. MTR maps were calculated from non-reformatted MT and REF datasets. A blurring Gaussian filter was applied in the MTR maps before segmentation measurements

Off-line reconstruction and post-processing was done in Matlab (The MathWorks, Inc., Natick, MA, USA). Two types of motion correction approaches were evaluated: (I) beat-to-beat 2D translational motion correction and (II) combined beat-to-beat 2D translational plus bin-to-bin non-rigid motion correction [22]. A scheme of the data flow is shown in Fig. 2a.

Beat-to-beat translational motion correction was performed by compensating the displacement in image space with a linear phase shift in *k*-space, using a property of Fourier transformations [23]:

1 $$ K_{{{\text{cor}}}} = K_{{{\text{acq}}}} {\text{e}}^{{2i\pi \vec{k} \cdot \vec{d}}} , $$

where ${K}_{\mathrm{a}\mathrm{c}\mathrm{q}}$ and ${K}_{\mathrm{c}\mathrm{o}\mathrm{r}}$ represent the acquired and corrected data, respectively, $\overrightarrow{k}$ is the *k*-space trajectory vector ${(k}_{x},{k}_{y})$ and $\overrightarrow{d}$ is the respiratory motion displacement ${(d}_{x},{d}_{y})$ in the left-to-right and foot-to-head directions, respectively, obtained from the iNAVs by normalized cross-correlation.

In the non-rigid motion correction framework, respiratory motion in the foot–head direction was derived from the iNAVs to assign the data into respiratory phases or bins (3–5 bins, maximum bin size 3.5 mm). Then, beat-to-beat 2D translation motion correction in the left-to-right and foot-to-head directions [according to Eq. (1)] was applied with respect to the centre of each bin, followed by iterative SENSE reconstruction to obtain an intra-bin motion-corrected 3D image for image of each respiratory phase so that 3D motion fields between respective respiratory phases (i.e. bins) could be obtained via non-rigid registration [24] of the 3D images. The parameters involved in the estimation of 3D motion fields include the weight of the penalty (i.e. in our case the bending energy) for an objective function based on normalized mutual information (using values as proposed by Modat et al. [24]) and the spacing or size of the deformation grid (in each of the three dimensions). Spacing was adjusted according to image resolution with an iterative experimental approach.

The motion fields, coil sensitivity maps, sampling trajectory and measured *k*-space data were incorporated in a motion-corrected generalized matrix description (GMD) [22, 25], whereby a motion-corrected 3D dataset was then obtained by solving the inverse problem using a conjugate gradient algorithm.

Motion correction was applied to the MT-prepared and REF datasets independently. In each, an end-expiration position iNAV was chosen as ‘reference’ for the cross-correlation procedure. After motion-corrected reconstruction, the MT-prepared and REF datasets were co-registered to correct any small misalignments [24]. MTR maps were calculated voxel-wise as:

2 $$\mathrm{M}\mathrm{T}\mathrm{R}= 100 \times \left(1- \frac{{I}_{\mathrm{M}\mathrm{T}}}{{I}_{\mathrm{R}\mathrm{e}\mathrm{f}}}\right),$$

where ${I}_{\mathrm{M}\mathrm{T}}$ and ${I}_{\mathrm{R}\mathrm{E}\mathrm{F}}$ are the signal intensity of the MT and REF datasets, respectively.

### Numerical simulations

Bloch simulations with a two-pool model of exchange were used to determine optimal MT preparation parameters. The evolution of the transverse and longitudinal magnetization of the free pool (${M}_{x,y}^{A}$, ${M}_{z}^{A}$, respectively) and bound pool (${M}_{x,y}^{B}$, ${M}_{z}^{B}$, respectively) under off-resonance irradiation with frequency ∆*F* and amplitude ${\omega }_{1}$ can be described by a modified set of Bloch equations [26]:

3 $$\frac{\partial {M}_{x}^{A}}{\partial \mathrm{t}}=-2\pi \Delta F{M}_{y}^{A}- \frac{{M}_{x}^{A}}{{T}_{2}^{A}}-{\mathfrak{I}(\omega }_{1}){M}_{z}^{A},$$

4 $$\frac{\partial {M}_{y}^{A}}{\partial \mathrm{t}}=2\pi \Delta {FM}_{x}^{A}- \frac{{M}_{y}^{A}}{{T}_{2}^{A}}+{\mathcal{R}(\omega }_{1}){M}_{z}^{A},$$

5 $$\frac{\partial {M}_{z}^{A}}{\partial \mathrm{t}}={\mathfrak{I}({\omega }_{1}){M}_{z}^{A}- \mathcal{R}(\omega }_{1}){M}_{y}^{A}+{R}_{A}\left({M}_{0}^{A}- {M}_{z}^{A}\right)-R{M}_{0}^{B}{M}_{z}^{A}+ R{M}_{0}^{A}{M}_{z}^{B},$$

6 $$\frac{\partial {M}_{z}^{B}}{\partial \mathrm{t}}={R}_{B}\left({M}_{0}^{B}- {M}_{z}^{B}\right)-\left({R}_{\mathrm{R}\mathrm{F}\mathrm{B}}+R{M}_{0}^{A}\right){M}_{z}^{B}+ R{M}_{0}^{B}{M}_{z}^{A},$$

where $\mathfrak{I}$ and $\mathcal{R}$ are the imaginary and real parts of ${\omega }_{1}$, respectively. The modification includes precession terms associated with the irradiation frequency ($\Delta F$), which are added in Eqs. (3) and (4), while terms dependent on the exchange rate ($R$) are added in Eqs. (5) and (6). ${R}_{A}$ and ${R}_{B}$ are the longitudinal relaxation rates of the free (*A*) and bound (*B*) pools, respectively.

Under off-resonance irradiation, the bound pool experiences direct saturation of its longitudinal magnetization. This is due to its extremely short ${ T}_{2}$, which causes negligible feedback with the transversal components. The saturation rate of the bound pool (${R}_{\mathrm{R}\mathrm{F}\mathrm{B}}$) is calculated as proportional to the RF power (${\omega }_{1}^{2}$) and the bound pool’s line absorption shape using an experimental super-Lorentzian approximation for semisolid systems [27]. In this model, the bound pool ${T}_{2}$ was fixed at ${T}_{2}^{B}=14$ μs for all simulations. The equilibrium magnetizations of the bound pool and free pool (${M}_{0}^{B}{,M}_{0}^{A}$) represent their size and therefore the ratio $PSR=\frac{{M}_{0}^{B}}{{M}_{0}^{A}+{M}_{0}^{B}}$ is the relative size of the bound pool [28, 29].

For a series of short and shaped off-resonance pulses, both ${\omega }_{1}$ and ${R}_{\mathrm{R}\mathrm{F}\mathrm{B}}$ are time dependent and no closed-form solution for the resulting magnetization can be found. Portnoy and Stanisz proposed a numerical approach [30] in which the RF pulse is split into small time intervals $\mathrm{d}t\approx 50$ µs, whereby its amplitude may be considered constant. The evolution of the net magnetization vector ${\overrightarrow{M}}_{A,B}\left(t\right)$ over a time $\mathrm{d}t$ is then solved as a linear problem and each transient solution propagates as the initial state for the next interval, until the end of the pulse is reached.

The MTR sequence was simulated using the described model to optimize its sensitivity to the pool size ratio (PSR) in myocardium-like tissue.

Simulated MT preparation parameters included bandwidth = 270 Hz, Sinc shape, length = 20.48 ms, delay between pulses = 1.5 ms, and pulse repetitions *n* = 20. Tissue parameters in myocardial simulations were performed using an exchange rate *R* of 50 Hz, ${T}_{1}^{A}$/${T}_{2}^{A}$ times of 1100/55 ms, and PSR of 15%, unless stated otherwise.

Simulations in myocardial-like tissue were performed over a range of Δ*F* (200–8000 Hz), MT pulse flip angles (360–900°), B0 inhomogeneity (from − 250 to 250 Hz), free pool relaxation times ($${T}_{1}^{A}$$=1000–1500 ms, $$ {T}_{2}^{A}$$=  30–80 ms), and exchange-related parameters (*R* = 20–70 Hz, PSR = 0–30%). A simulated constant heart rate of 65 beats per minute (bpm) was used. The signal was measured from the 15th echo (the sampling of the *k*-space centre) during the fifth heartbeat, to ensure *pseudo* steady-state conditions. Two different imaging modules were simulated: (I) SPGR with sequence parameters including TR/TE = 3.8/1.6 ms and FA = 15°, (II) bSSFP with sequence parameters including TR/TE = 3.2/1.4 ms and FA = 70°. A single-pool (no exchange) model was assumed during the pulses of the imaging module.

### Phantom study

Agar samples with different concentrations (1%, 2%, 2.5% and 5% agar in water) were prepared and used to validate the signal model and evaluate the performance of the proposed sequence in the presence of different MT exchange parameters and in the absence of motion. The proposed sequence was acquired using SPGR (TR/TE = 3.8/1.6 ms, FA = 15°, BW = 401 Hz/px, field of view (FoV) = 300 × 300 × 112 mm^3^, resolution = 1.4 × 1.4 × 4 mm^3^, number of profiles/excitations = 30) and bSSFP (TR/TE = 3.2/1.4 ms, FA = 70°, BW = 925 Hz/px). The MT pulse parameters were the same as described for the simulations. Data were acquired over a wide range of MT pulse flip angles (360–900˚) and off-resonance frequencies (400–8000 Hz).

### Animal study

Animal experiments were conducted in Landrace pigs (*n* = 3) at the Institut de Chirurgie Guidée par l’image (IHU), Strasbourg, France. The pigs were scanned in vivo 6 weeks after undergoing left anterior descending artery occlusion for 3 h, using the procedure described by Tschabrunn et al. [31]. Each animal was scanned with the proposed sequence using the following imaging parameters: coronal orientation, FoV = 300 × 300 × 108 mm^3^, resolution = 1.7 × 1.7 × 6 mm^3^, SPGR (TR/TE = 3.8/1.76 ms, FA = 15°, BW = 535 Hz/px). MT preparation pulses had bandwidth, shape, length, and delay between pulses as described in the phantom study. MT off-resonance frequency was ∆*F* = 1500 Hz, pulse repetitions *n* = 10 and flip angle = 720°.

A 3D bSSFP LGE dataset was obtained following 10–20 min after injection of the contrast agent (Gadovist, Bayer, Germany). Imaging parameters included coronal orientation (matching the MTR scan), FoV = 200 × 260 × 105mm^3^, resolution = 1.2 × 1.2 × 1.2mm^3^, and bSSFP (TR/TE = 3.2/1.58 ms, FA = 90°, BW = 930 Hz/px).

Respiratory motion was very limited due to ventilation and did not affect the heart position, therefore no motion correction/compensation procedures were applied.

Scar, remote and total myocardium area were manually segmented in LGE images by a single observer (Lopez) with experience in cardiac LGE (approximately 3 years). Automated segmentation was not attempted. MTR scar volume was determined by thresholding in each separate animal as follows: (1) after manually selecting a remote (myocardium) area in the MTR map, mean and standard deviations (σ) were calculated; (2) a mask (*m*1) of the entire myocardium was manually drawn using the MT-weighted dataset (intrinsically co-registered to the MTR map); (3) finally, MTR voxels under the threshold mean*-*2*σ* and within *m*1 were automatically selected as scar volume. The overlap was defined as the number of coincidences between LGE and MTR scar voxels, divided by the total number of LGE scar voxels:

7 $$\mathrm{O}\mathrm{v}\mathrm{e}\mathrm{r}\mathrm{l}\mathrm{a}\mathrm{p}=100 \times \frac{\#\left\{{\mathrm{L}\mathrm{G}\mathrm{E}}_{\mathrm{s}\mathrm{c}\mathrm{a}\mathrm{r}}\cap {\mathrm{M}\mathrm{T}\mathrm{R}}_{\mathrm{s}\mathrm{c}\mathrm{a}\mathrm{r}}\right\}}{\#{\mathrm{L}\mathrm{G}\mathrm{E}}_{\mathrm{s}\mathrm{c}\mathrm{a}\mathrm{r}}}.$$

The false positive percentage was calculated using the number of coincidences between MTR scar voxels and LGE remote voxels:

8 $$\mathrm{fa}\mathrm{l}\mathrm{s}\mathrm{e} \mathrm{p}\mathrm{o}\mathrm{s}\mathrm{i}\mathrm{t}\mathrm{i}\mathrm{v}\mathrm{e}=100 \times \left(1- \frac{\#\left\{{\mathrm{L}\mathrm{G}\mathrm{E}}_{\mathrm{r}\mathrm{e}\mathrm{m}\mathrm{o}\mathrm{t}\mathrm{e}}\cap {\mathrm{M}\mathrm{T}\mathrm{R}}_{\mathrm{s}\mathrm{c}\mathrm{a}\mathrm{r}}\right\}}{\#{\mathrm{L}\mathrm{G}\mathrm{E}}_{\mathrm{r}\mathrm{e}\mathrm{m}\mathrm{o}\mathrm{t}\mathrm{e}}}\right)$$

### Healthy subjects study

Ten healthy human subjects (29 ± 3 years, two males) were recruited for a proof-of-concept evaluation of the proposed sequence with translational and non-rigid motion corrections. Each subject was imaged twice, using bSSFP (TR/TE = 3.2/1.4 ms, FA = 70°, BW = 925 Hz/px) and SPGR (TR/TE = 3.8/1.6 ms, FA = 15°, BW = 500 Hz) imaging modules. To avoid artefacts observed at the liver interface, the MT preparation was of equivalent power deposition but higher off resonance frequency than that of the animal study (see Supplementary data, Fig. 1). The modified MT preparation parameters were: MT flip angle = 800˚, ΔF = 3000 Hz and pulse repetitions *n *= 20. Other imaging parameters included: 3D coronal orientation, FoV = 300 × 300 × 112 mm^3^, acquired resolution = 1.4 × 1.4 × 4 mm, reconstructed resolution = 1.4 mm^3^, 100% respiratory gating efficiency, and nominal scan time of 9:02 min at 60 bpm.

A data flow scheme for the image analysis pipeline is shown in Fig. 2b. All datasets were reformatted in short axis orientation, using tri-linear interpolation. A 2D Gaussian filter was applied (kernel size 5 pixel, *σ* = 3 pixel) on every slice of the MTR maps to reduce the noise and improve visualization.

Manual segmentation was performed for the three left ventricular slices (basal, mid and apical) using the American Heart Association (AHA) 16-segment model of myocardial segmentation [32].

The SPGR and bSSFP variants of the sequence were compared using the following metrics: (I) average global MTR and average MTR per segment; (II) spatial variation of MTR in each segment, calculated as the ratio between the standard deviation and the mean MTR (expressed as a percentage); (III) contrast ratio (CR) between blood MTR and myocardial MTR.

MTR mean values and spatial variability were also calculated in regions of interest (ROIs) in the liver and pectoral muscle to compare with myocardial values and to assess the impact of different types of motion in the spatial variability of MTR.

Based on the bSSFP results in simulations and phantom experiments, the quality of coronary vein visualization was evaluated in the SPGR datasets only, using vessel sharpness (VS) and contrast-to-noise ratio (CNR) measurements. Translational vs non-rigid motion correction approaches were compared using a reformatted 2D representation of the 3D course of the cardiac veins [33]. VS was calculated for the posterior branch of the cardiac vein (PCV) and the great cardiac vein (GCV) from the reformatted images. VS was defined as the average intensity gradient along the edges normalized to the signal intensity of the centre of the vessel. Contrast-to-noise-ratio (CNR) between blood and myocardium was obtained from the reformatted images using:

9 $${\mathrm{C}\mathrm{N}\mathrm{R}}_{\mathrm{M}\mathrm{T}}=\frac{{S}_{\mathrm{B}\mathrm{l}\mathrm{o}\mathrm{o}\mathrm{d}}- {S}_{\mathrm{M}\mathrm{y}\mathrm{o}}}{{\upsigma }_{\mathrm{L}\mathrm{u}\mathrm{n}\mathrm{g}\mathrm{s}}},$$

where ${S}_{\mathrm{B}\mathrm{l}\mathrm{o}\mathrm{o}\mathrm{d}}$ and ${S}_{\mathrm{M}\mathrm{y}\mathrm{o}}$ are the mean signal intensity in blood and myocardium ROI, respectively, and ${\sigma }_{\mathrm{L}\mathrm{u}\mathrm{n}\mathrm{g}\mathrm{s}}$ is the standard deviation in a small region of the lungs with no vessels.

## Results

### Numerical simulations and phantom study

**Fig. 3:**
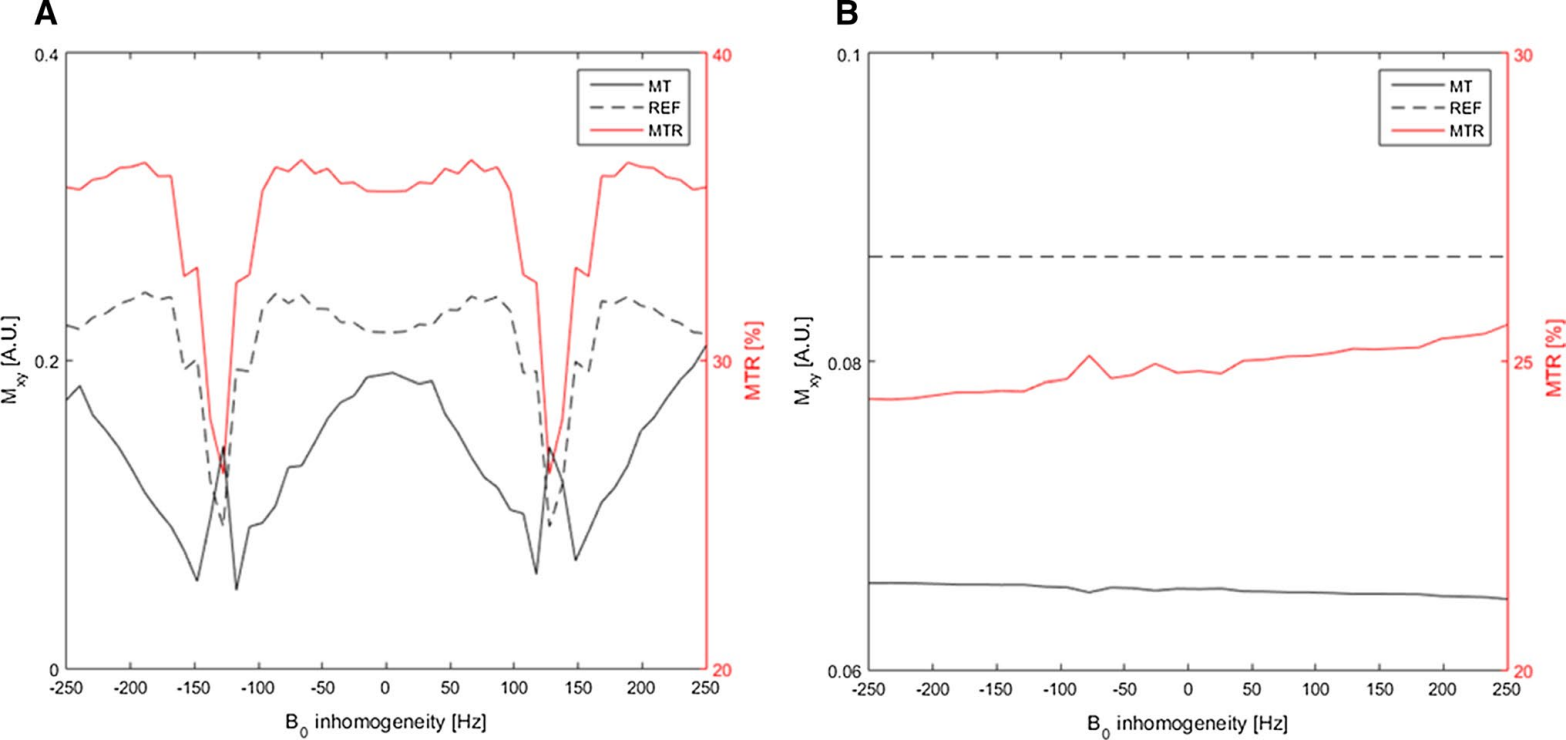
**a** Simulation of signal (Mxy) of the proposed sequence in myocardium-like tissue with balanced steady-state free precession, as a function of B0 inhomogeneity. Signal from the MT and REF acquisitions (left axis) showed ‘stop’ bands (1/2TR) approximately at ± 150 Hz, while the MTR (right axis) showed a maximum reduction of up to 8% at B0 <  ± 150 Hz compared to B0 = 0. **b** Simulations as described in **a**, except with an SPGR imaging module instead. The robustness of SPGR is shown in the flat profile obtained for the REF dataset. MTR signal is only marginally affected (< 3%) throughout the B0 inhomogeneity range that was in the range of ± 200 Hz

Bloch-simulated MTR sensitivity to B0 inhomogeneity in myocardium-like tissue with both imaging modules (bSSFP vs SPGR) is shown in Fig. 3a and b, respectively. SPGR yielded an almost flat MTR response with less than 3% variation across the studied B0 range. The bSSFP module led to generally higher MTR values, but had a strong sensitivity to B0 inhomogeneity with approximately 20% variation towards the 1/2TR bands at ± 150 Hz.

**Fig. 4:**
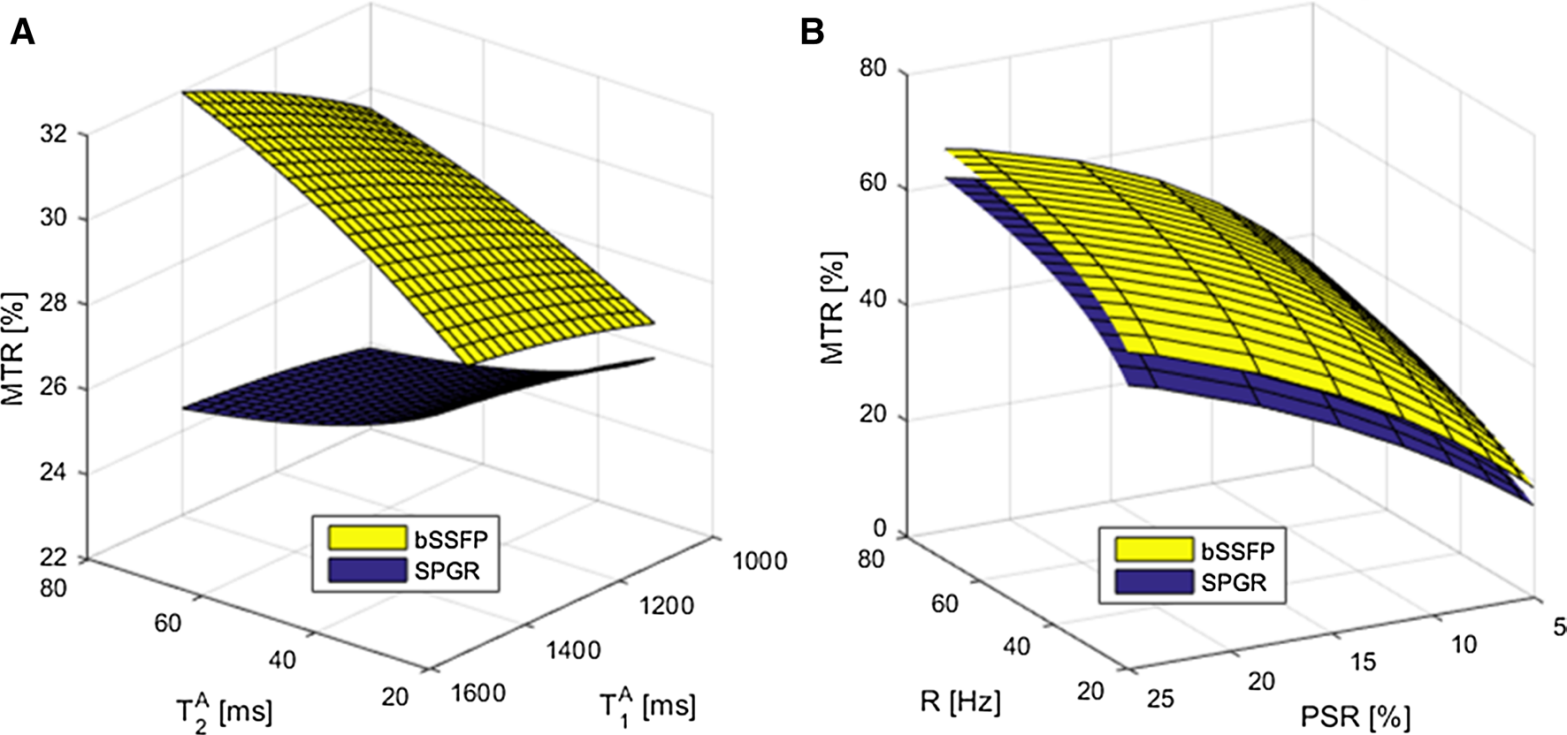
**a** MTR sensitivity to the free pool relaxation rates, ${T}_{1}^{A}$ and ${T}_{2}^{A}$, comparing bSSFP and SPGR imaging modules. SPGR showed a flatter profile with MTR variations less than 2% across a common range of relaxation rates. **b** MTR sensitivity to the exchange rate (*R*) and the pool size ratio (PSR) for bSSFP and SPGR. Both imaging modules showed similar increase in MTR as a function of PSR, as well as a more moderate increase in MTR as a function of *R*

MTR sensitivity curves with respect to the relaxation rates and exchange-related parameters are shown in Fig. 4, both for SPGR and bSSFP imaging modules. MTR sensitivity to ${T}_{1}^{A}$ and ${T}_{2}^{A}$ using SPGR was lower than bSSFP (${T}_{2}^{A}$: < 2.3% *vs* < 4.3%; ${T}_{1}^{A}$: < 0.7% *vs* < 2.5%, respectively) within the simulated range of myocardial relaxation rates (Fig. 4a). MTR values were found to increase as a function of PSR and *R* (Fig. 4b). *PSR* proved to be the major contributor to MTR, showing a near-linear dependency within the range associated with human muscle (PSR = 5–15%). MTR dependency of the exchange rate was slowly increasing (less than 7%) within a range of *R* = 20–70 Hz. The imaging module (bSSFP vs. SPGR) was found to have little impact on the shape of MTR as a function of *R* and PSR, as observed in Fig. 4b.

**Fig. 5:**
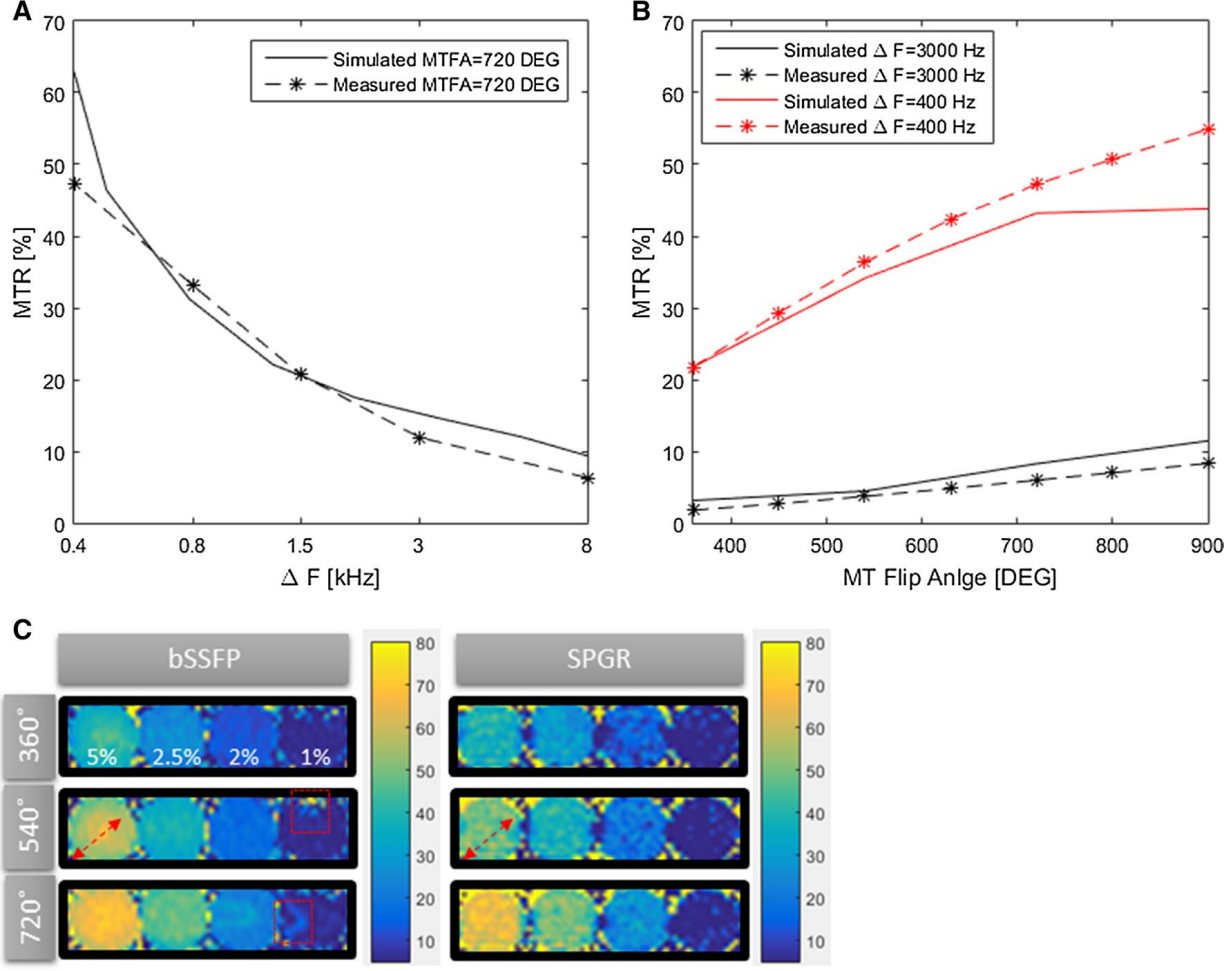
**a** MTR dependency as a function of the off-resonance frequency (Δ*F*) showed strong dependency at low Δ*F*, i.e. Δ*F* < 1.5 kHz, for a phantom made of 2.5% concentration of agar. **b** MTR as function of MT flip angle showed steeper slope for low Δ*F* (400 Hz) than that of higher Δ*F* (3000 Hz) with nonlinear dependency at high flip angles (> 800˚), for a phantom made of 2.5% concentration of agar. Measured and simulated data were in good agreement in **a** and **b**. **c** MTR images of the four agar-based samples (1, 2, 2.5, and 5% concentration) with bSSFP and SPGR imaging modules using different MT flip angles and fixed Δ*F* = 400 Hz. Red dashed arrows show an intensity gradient from centre to periphery in the bSSFP (not observed in SPGR). Likewise, red dashed squares show other artefacts seen only in the bSSFP acquisition

The effects of ∆*F* and MT flip angle on MTR values using SPGR on a 2.5% agar sample are shown in Fig. 5a and b, respectively. MTR values showed a strong non-linear frequency dependency for low *∆F* (< 1 kHz) and a less strong dependency at higher *∆F*. The MTR dependency on the flip angle was near linear for flip angles < 800° with a stronger flip angle dependency for lower off-resonance frequencies. The phantom measurements and simulated data were in good agreement over a wide range of *∆F* (> 0.4 Hz and < 8 kHz) and MT flip angles (> 360° and < 800°). MTR maps obtained with bSSFP and SPGR using three different flip angles and fixed *∆F* = 400 Hz are shown in Fig. 5c. The MTR maps acquired with bSSFP showed multiple artefacts, likely associated with B0 inhomogeneity. The SPGR-based MTR maps were homogenous throughout but noisier than bSSFP.

### Animal study

**Fig. 6:**
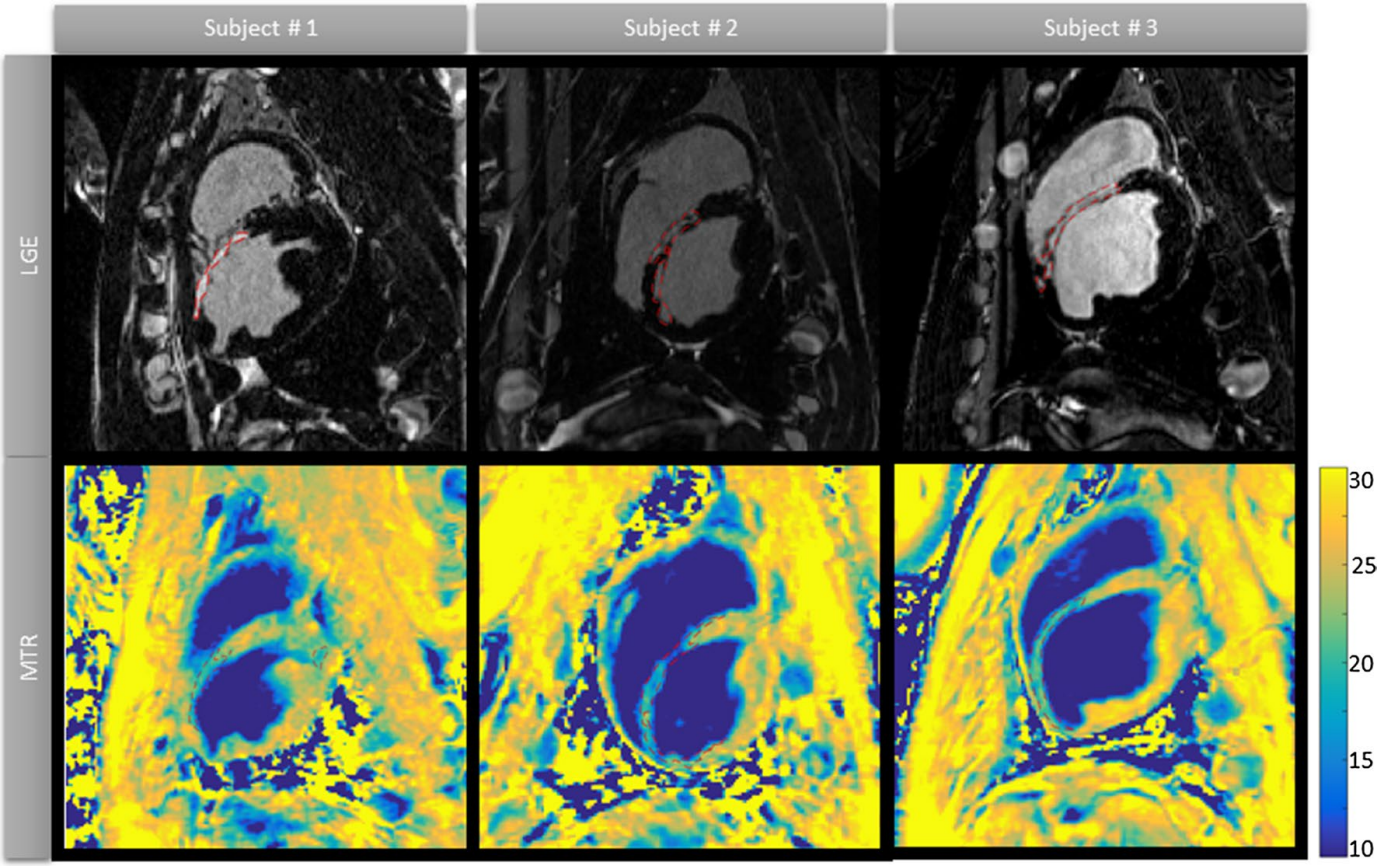
Co-registered coronal slices comparing MTR maps with LGE images in three pigs, 6 weeks after left anterior descending artery occlusion. In all pigs, the MTR maps show significant reduction in areas that overlap with signal enhancement in the LGE images, allowing clear localization of myocardial scar from the contrast-free MTR maps alone

MTR decrease was found in regions associated with scar and was in good agreement with the presence of LGE in all three animals. MTR maps allowed accurate localization and visualization of the scar (see Fig. 6). Mean MTR values in remote myocardium were 25.1 ± 1.7%, while mean MTR values in scar were 16.8 ± 1.9%. Voxel-to-voxel comparison with LGE showed a mean overlap of 62.5 ± 19.2% and a mean false positive percentage of 9.6 ± 6.0% over all animals (*n* = 3).

### Healthy subjects

The bSSFP module yielded higher CNR than SPGR (13.9 ± 1.2, vs. 8.1 ± 0.7, *p* < 0.05) in the MT-prepared datasets. CR measurements in the MTR maps (between blood and myocardium) showed no statistically significant difference between the two imaging modules [bSSFP: 2.50 ± 0.16 vs. SPGR: 2.48 ± 0.32, *p* = not statistically significant (NS)].

**Fig. 7:**
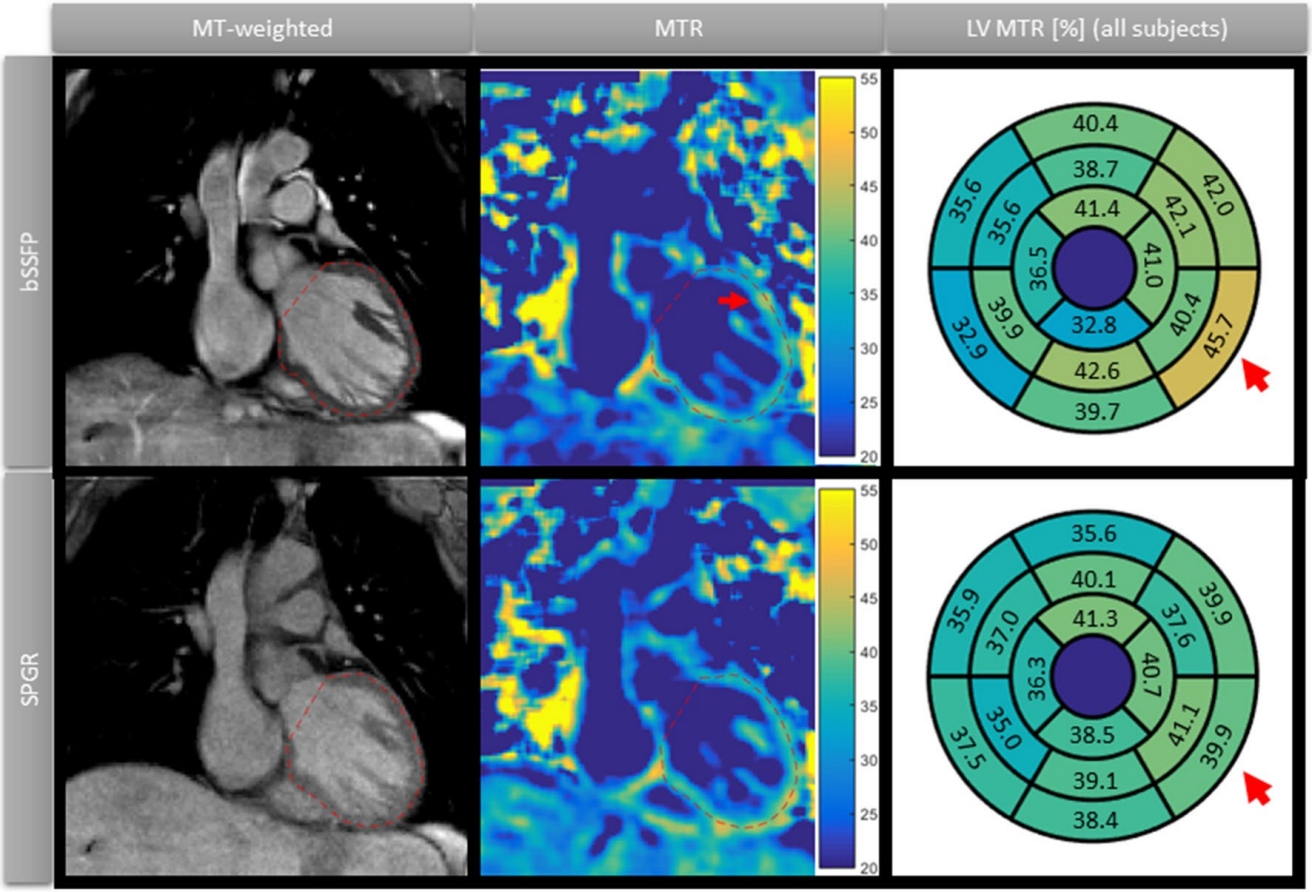
Left and middle columns show a coronal slice in a representative healthy subject, comparing the MT-prepared dataset with the MTR map. bSSFP and SPGR imaging modules are shown in each row. The MTR map looks more homogenous with SPGR than with bSSFP, where there is an artefact in the lateral region (red arrow). The right column shows MTR mean values per segment of the American Heart Association (AHA) 16-segment model, averaged over the entire cohort of healthy subjects (*n* = 10). The SPGR imaging module showed reduced intersegment variation compared to bSSFP (red arrows)

Representative MTR maps and myocardial segment mean values across all subjects using SPGR and bSSFP are shown in Fig. 7. The MTR maps acquired with bSSFP demonstrated increased MTR values towards the lateral and inferior regions. This may be due to B0 inhomogeneity at the lung–myocardium interface. A typical artefact in this region is shown in Fig. 7 (middle column). Whole heart myocardium MTR averaged over all subjects and obtained after translational motion correction was 39.2 ± 1.9% using bSSFP and 37.0 ± 2.1% using SPGR. Spatial variation of MTR was 19.3 ± 2.9% vs. 15.7 ± 2.1% (*p* = 0.01) for bSSFP vs. SPGR, respectively. SPGR led to reduced MTR variability between segments compared to bSSFP (8.0 ± 2.3% vs 10.3 ± 1.9%, *p* = 0.07), as seen in Fig. 7 (right column). Mean MTR values and spatial variation did not show statistically significant difference between translational and non-rigid motion correction, as shown in Table 1.

**Table 1:** Myocardium MTR mean values and spatial variation (SD/mean)

Mean MTR (%)			*p*
Readout/MC	Translational	Non-rigid	
bSSFP	39.2 ± 1.9	39.1 ± 1.5	n.s
SPGR	37.0 ± 2.1	37.0 ± 1.8	n.s
*p*	< 0.01	< 0.01	

Spatial variation			*p*
Readout/MC	Translational	Non-rigid	
bSSFP	19.3 ± 2.9	19.4 ± 3.2	n.s
SPGR	15.7 ± 2.1	15.7 ± 2.5	
*p*	< 0.01	< 0.01	

*MC* motion correction, *SPGR* spoiled gradient echo, *bSSFP* balanced steady-state free precession, *n.s.* non-statistically significant

MTR values in pectoral muscle showed significantly reduced spatial variability compared to myocardium: MTR = 43.8 ± 1.7 (spatial variability: 9.5 ± 3.4%, *p* < 0.01) and MTR = 41.0 ± 2.4 (spatial variability: 5.8 ± 3.6%, *p* < 0.01), for bSSFP and SPGR, respectively. Liver MTR measurements showed similar spatial variability compared to myocardium, with MTR = 32.3 ± 2.9 (spatial variability: 18.4 ± 4.3%, *p* = 0.4) and MTR = 29.6 ± 3.5 (spatial variability: 15.5 ± 0.8%, *p* = 0.7), for bSSFP and SPGR, respectively. One reason for this similarity may be that the liver and the heart experience similar extent of respiratory motion.

**Fig. 8:**
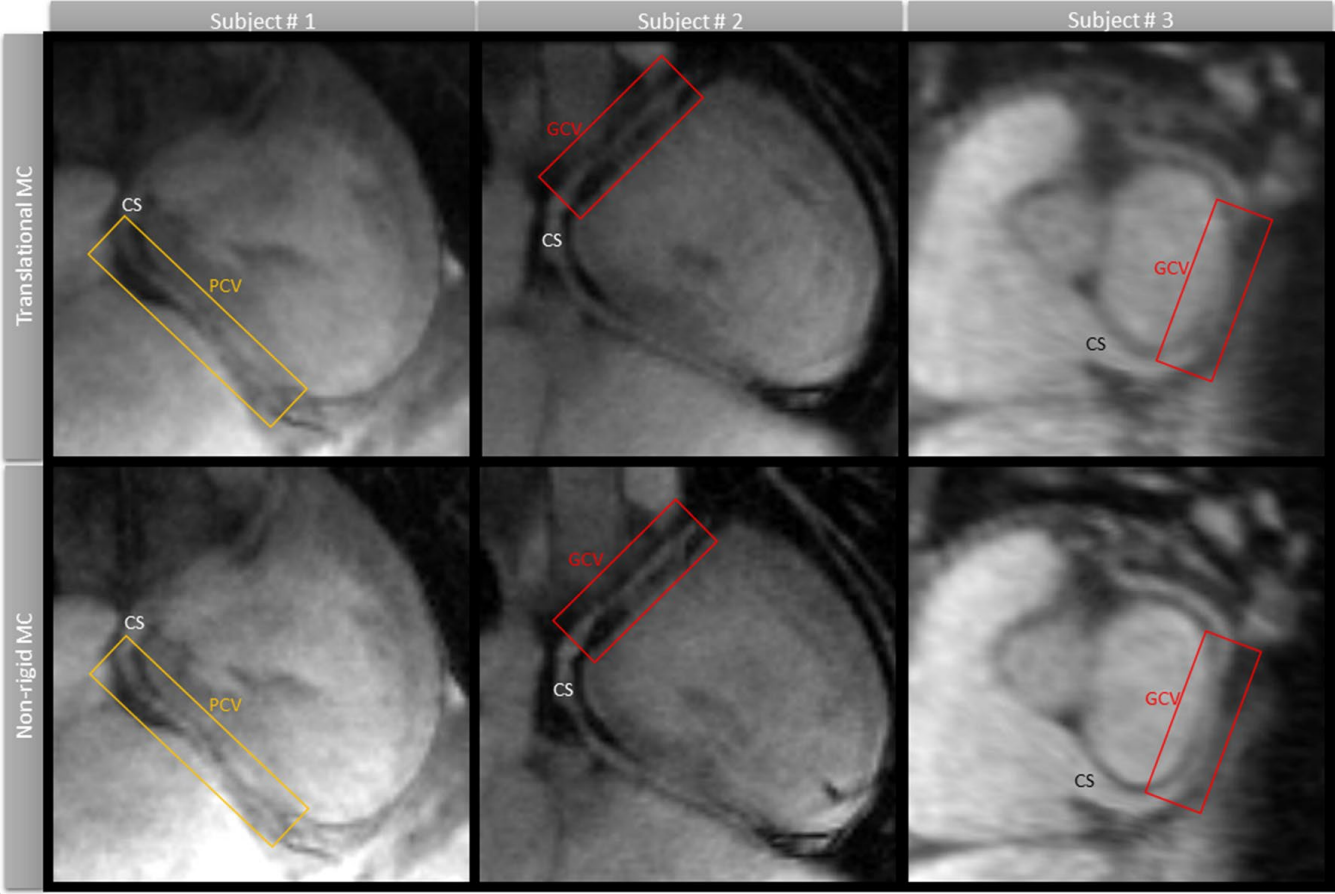
Main coronary veins and structures such as coronary sinus (CS), the great cardiac vein (GCV) and the posterior branch of the cardiac vein (PCV) are shown in three different subjects, comparing the two motion correction (MC) approaches. The non-rigid MC improves the visualization with respect to the translational MC in all cases, recovering blurred features and increasing vessel sharpness

The use of translational motion correction allowed good depiction of the PCV and GCV. The non-rigid motion correction improved visualization of the entire cardiac anatomy with respect to the translational motion correction. This was observed in an increase in vessel sharpness in the GCV (40.8 ± 2.0% vs 32 ± 2.5%, *p* < 0.05) and the PCV (34.1 ± 3.2% vs 30.0 ± 1.7%, *p* < 0.05). Three examples from representative subjects are shown in Fig. 8.

## Discussion

A motion-corrected free-breathing 3D whole heart MTR mapping sequence was developed and successfully validated in phantoms, healthy subjects and pigs. MTR maps allowed the localization of myocardial fibrosis without the injection of a contrast agent in a pig model of myocardial infarction. Significantly lower MTR values were found in areas that corresponded with hyperintense signal on LGE. In human healthy subjects, MT-prepared images reconstructed with non-rigid motion correction enabled the visualization of the cardiac veins with vessel sharpness between 30 and 40% along the main cardiac veins.

MTR values found in an agar-based phantom and healthy subjects were overall consistent with Bloch simulations using a two-pool exchange model, which suggested increased MT effects associated with increased pool size ratio. Though a small discrepancy was observed in Fig. 5b, at low off-resonance frequency (Δ = 400 Hz) and high flip angles, this was likely due to residual on-resonance saturation of the free pool not being included in the model, which seems to “inflate” the measured MTR increasingly as the flip angle increases.

In accordance with the simulations, MTR myocardial values obtained with an SPGR imaging module had less spatial variability compared to those obtained with bSSFP. This result was in agreement with a study of coronary vein imaging [17] that found that SSFP sequences were more prone to artefacts than SPGR, especially in regions surrounding deoxygenated blood (e.g. coronary veins), likely because of the spread of the resonant frequency. A similar effect was discussed in a study of coronary artery imaging [34], which highlights the challenges in finding a good shimming solution for the heart due to, at least in part, cardiac and respiratory motion, blood flow, chemical shift, and susceptibility variations at air–tissue interfaces.

In healthy subjects, the spatial variability of myocardial MTR was higher than in static tissues such as pectoral muscle. This suggests that respiratory motion might have an impact on spatial variability of MTR and that residual respiratory motion may have been present in the MTR maps. While non-rigid respiratory motion correction was shown to improve vessel sharpness in the MT-prepared dataset, the standard deviation of myocardial MTR was not significantly improved compared to translational motion correction only. The use of more advanced navigation techniques such as 3D iNAVs [35, 36] may help to tackle unaccounted respiratory motion. Error or bias associated with non-rigid motion within a respiratory phase may be reduced upon optimization of the size and number of bins which, in this study, were based on values previously obtained by our research group [20] for the visualization of coronary vessels. The differences between bSSFP and SPGR also suggest that field inhomogeneity may play a role in increased spatial variability. The individual contributions of residual motion and field inhomogeneity to MTR spatial variability may be assessed with an MT-prepared breath-held single-shot sequence in future studies.

Six-week-old scar in pigs was easily visualized and was characterized by a decrease in MTR. These results are in good agreement with previous studies which reported decreased MTR values in porcine heart 4 weeks after MI [37] and acute MI in humans [18, 38]. In the latter setting, it was hypothesized that the presence of oedema leads to an increase in the absolute size of the free pool and thus a relative decrease in the MT effect and MTR. In a post-mortem study that compared MTR with histology [39], Crooijmans et al. found a relationship between increased MTR and fibrotic tissue, as well as a relationship between decreased MTR and inflammatory response, due to granulocyte infiltration or oedema. In another study in pigs performed 6 weeks after coronary occlusion, Dhanjal et al. showed massive cellularity (stromal cells such as fibroblasts and myofibroblasts), vascularity and heterogeneity in the scarred zones [40], which might help to explain the significant decrease in MTR associated with scar in our experiments. A histopathology experiment in conjunction with MTR imaging could help to support this hypothesis in future studies.

Another contrast-free approach to probe the spins with restricted motion is the T1-ρ technique, which explores spin–lattice relaxation within the rotating frame. Multiple studies have found increased T1-ρ in knee cartilage in patients with osteoarthritis due to the loss of proteoglycans and collagen (type II) integrity [41, 42], i.e. a decrease in the size of the macromolecular pool. However, 8 weeks post-MI, pigs also showed increased T1-ρ values associated with collagenous (mostly type I and III) scar [43, 44] where the size of the extracellular matrix (and macromolecular pool) was expected to have increased. In a study with acute MI patients, Muthupillai et al. suggested that changes in T1-ρ in the infarct zone were also associated with the loss in macromolecular content due to myocyte death [45].

At least two competing processes that may increase or decrease the size of the bound pool are at play in cardiac remodelling after MI, namely, the growth of the extracellular matrix and the death of protein-rich cardiomyocytes, respectively. The presence of connective tissue or oedema within the fibrotic scar may also increase the size of the free pool. Our results in 6-week-old (post MI) scar showed that MTR was significantly decreased, suggesting that the proposed MT preparation might be more sensitive to myocyte death rather than the known increase in collagen content associated with chronic infarct [46].

This interpretation would greatly benefit from data at various stages of the cardiac remodelling process to assess the specificity and utility of the technique in clinical practice. In an experimental mouse model, Vandsburger et al. reported increasing MT effects after induced MI from day 7 to 21 with a cine CEST technique [47]. To the best of our knowledge, there are no longitudinal studies in large animals that show the evolution of MTR or MT effects along the time course of the cardiac remodelling process beyond 6–8 weeks after MI.

### Limitations

The conflation of bound pool size, free pool size, exchange rate and all the relaxation rates into a single parameter, i.e. the MTR, may hinder the applicability of the technique within clinical settings, where oftentimes the aetiology of the scar may be unknown or multi-pronged. In these cases, a quantitative approach using a multi-parameter MT model could be more appropriate.

The number of off-line post-processing steps and setup parameters may slow down widespread clinical adoption; however, more automated or end-product frameworks that apply many of these same techniques are currently being developed by the present research group and others.

The small number of pigs used in this study was a limitation; however, the results showed consistency across all three cases. Other limitations included the relatively long scan time and anisotropic resolution in the human subjects. The anisotropic resolution affected image quality, specifically when reformatting to orientations other than coronal. The resolution was compromised to maintain a short scan time (< 10 min) that was clinically appealing. Future work might benefit from isotropic resolution paired with an accelerated acquisition framework.

## Conclusion

We have developed a non-contrast high-resolution 3D sequence that allows calculation of magnetization transfer ratio (MTR) maps for the assessment of myocardial scar and visualization of cardiac veins across the whole heart. Good agreement was found between MTR and LGE in a small cohort of pigs with 6-week-old scar. Further studies are warranted to fully characterize the potential of MT for imaging of myocardial fibrosis.

## Electronic supplementary material

Below is the link to the electronic supplementary material.

Supplementary file1 (DOCX 1552 kb)
