# Supplementary material for: Contrast-free high-resolution 3D magnetization transfer imaging for simultaneous myocardial scar and cardiac vein visualization
Source: MAGMA. 2020 Feb 20;33(5):627–40. doi: 10.1007/s10334-020-00833-9 (PMC7502043; doi:10.1007/s10334-020-00833-9)
Supplement: Supplementary file 1 — Supplementary file1 (DOCX 1552 kb) [file 10334_2020_833_MOESM1_ESM.docx]

**Contrast-free high-resolution 3D magnetization transfer imaging for simultaneous myocardial scar and cardiac vein visualization**

Karina López^1^, Radhouene Neji^1,2^, Rahul K. Mukherjee^1^, John Whitaker^1^, Alkystis Phinikaridou^1^, Reza Razavi^1^, Claudia Prieto^1^, Sébastien Roujol^1^, René Botnar^1^

^1^School of Biomedical Engineering and Imaging Sciences, King’s College London, UK

^2^MR Research Collaborations, Siemens Healthcare Limited, Frimley, UK

Corresponding author:

Karina Lopez,

School of Biomedical Engineering and Imaging Sciences, King’s College London, 3rd Floor Lambeth Wing, St Thomas’ Hospital, London, SE1 7EH,

Tel: +44(0)20 718 88299, email: karina.lopez@kcl.ac.uk

**SUPPLEMENTARY DATA**

*Optimization of MT preparation protocol in humans.*

In two human subjects, the MT preparation protocol used in animals (ΔF=1500 Hz, FA=720˚, repetitions=10, protocol 1) was compared against a second protocol (ΔF=3000 Hz, FA=800˚, repetitions=20, protocol 2), in order to investigate the impact of B0 inhomogeneity in myocardial MTR. The two preparation protocols had equivalent average RF power deposition. The imaging module was SPGR (TR/TE=3.8/1.6ms, FA=15°, BW=500 Hz).

The images acquired with protocol 1 showed a number of artifacts that either did not appear or were less significant in protocol 2’s images, as shown in two different cases in Figure 1. The artefacts observed in the myocardium with protocol 1 were typically associated with the liver interface.

The results suggested that B0 inhomogeneity may affect the accuracy of MTR in the myocardium when using protocol 1, possibly due to a strong gradient of magnetic susceptibility at the myocardium-liver interface. For this reason, protocol 2 was used as MT preparation in all further human studies.

*MTR snapshots of every subject in the healthy volunteer cohort*

An MTR coronal slice is shown for each of the subjects in the healthy volunteers cohort not shown in the main manuscript (n=9) in Figure 2. No acquired datasets in this cohort were discarded.


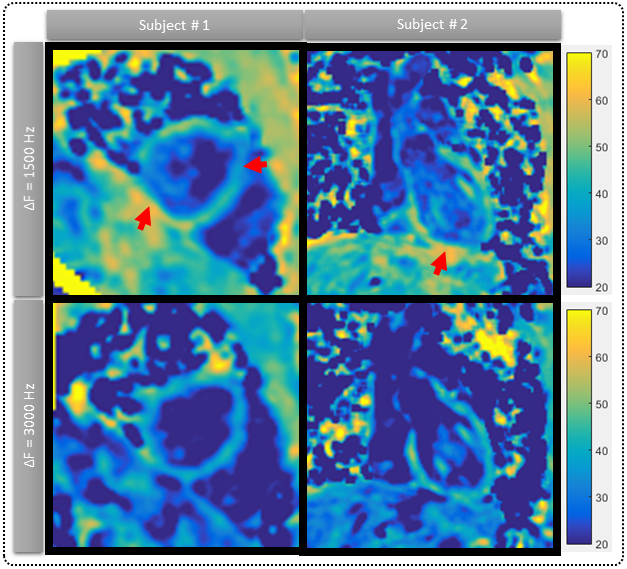


Figure 1. Comparison of MTR maps for two MT preparations with equivalent power deposition but different ΔF (ΔF =1500 or 3000 Hz) for two healthy subjects in short-axis (subject 1) and coronal (subject 2) views. The preparation with ΔF=1500 Hz shows areas of increased MTR associated with the myocardium-liver interface (red arrows).


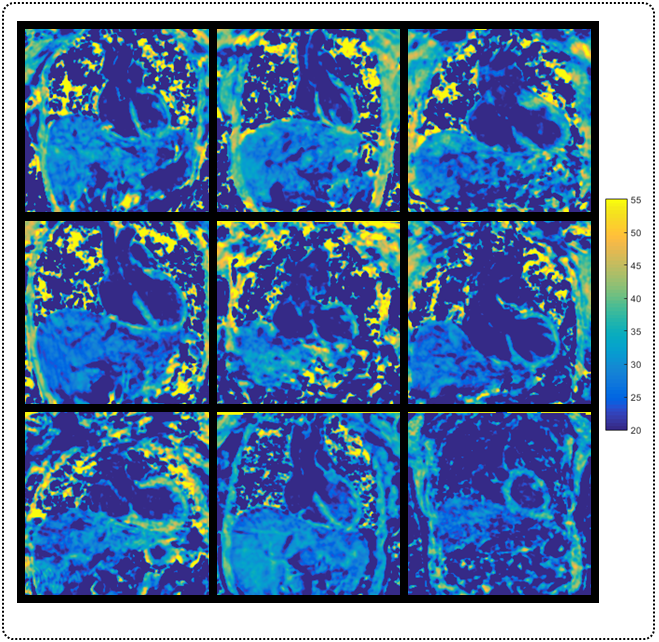


Figure 2. A Coronal slice of the MTR map of each of the 10 subjects of the healthy volunteer cohort which were not shown in the main manuscript, i.e., 9 subjects. No significant outliers were found.
